# The effects of a preoperative multidisciplinary conference on outcomes for high-risk patients with challenging surgical treatment options: a retrospective study

**DOI:** 10.1186/s12871-021-01257-1

**Published:** 2021-02-06

**Authors:** Masayoshi Koike, Mie Yoshimura, Yasushi Mio, Shoichi Uezono

**Affiliations:** Department of Anesthesiology, Jikei University School of Medicine, 3-25-8 Nishi-shinbashi, Minato-ku, Tokyo, 105-8461 Japan

**Keywords:** Preoperative multidisciplinary conference, Challenging surgical treatment option, Patient safety, Shared decision making

## Abstract

**Background:**

Surgical options for patients vary with age and comorbidities, advances in medical technology and patients’ wishes. This complexity can make it difficult for surgeons to determine appropriate treatment plans independently. At our institution, final decisions regarding treatment for patients are made at multidisciplinary meetings, termed High-Risk Conferences, led by the Patient Safety Committee.

**Methods:**

In this retrospective study, we assessed the reasons for convening High-Risk Conferences, the final decisions made and treatment outcomes using conference records and patient medical records for conferences conducted at our institution from April 2010 to March 2018.

**Results:**

A total of 410 High-Risk Conferences were conducted for 406 patients during the study period. The department with the most conferences was cardiovascular surgery (24%), and the reasons for convening conferences included the presence of severe comorbidities (51%), highly difficult surgeries (41%) and nonmedical/personal issues (8%). Treatment changes were made for 49 patients (12%), including surgical modifications for 20 patients and surgery cancellation for 29. The most common surgical modification was procedure reduction (16 patients); 4 deaths were reported. Follow-up was available for 21 patients for whom surgery was cancelled, with 11 deaths reported.

**Conclusions:**

Given that some change to the treatment plan was made for 12% of the patients discussed at the High-Risk Conferences, we conclude that participants of these conferences did not always agree with the original surgical plan and that the multidisciplinary decision-making process of the conferences served to allow for modifications. Many of the modifications involved reductions in procedures to reflect a more conservative approach, which might have decreased perioperative mortality and the incidence of complications as well as unnecessary surgeries. High-risk patients have complex issues, and it is difficult to verify statistically whether outcomes are associated with changes in course of treatment. Nevertheless, these conferences might be useful from a patient safety perspective and minimize the potential for legal disputes.

## Background

Surgical procedures have become more sophisticated and complex as a result of advances in medical technology [1]. Minimally invasive surgery is more common [1, 2], and indications for surgery have expanded to include the elderly and patients with severe medical comorbidities [3, 4]. Patients’ and their families’ wishes and values are diverse. The concept of death with dignity has become popular, and patients are more likely to expect treatments based on their individual preferences and wishes. Surgeons therefore need to consider medical as well as these nonmedical issues when developing an optimal treatment plan. This can be difficult to accomplish independently.

Anesthesiologists typically assess a patient’s condition and plan perioperative care in accordance with the surgeon’s plan [5]. However, the introduction of novel surgical procedures can lead to confusion and interfere with anesthesiology plans. Even for a minimally invasive surgical procedure, the invasiveness of anesthesia might not be minimal, depending on the patient’s condition [6]. For patients with numerous comorbidities, the anesthesiologist might feel that the risk of serious perioperative complications is disproportionately high compared to the benefit that the patient might obtain from the surgery. Estimating the perioperative risk of complications with a reproducible score, such as that available from the American College of Surgeons National Surgical Quality Improvement Program (ACS NSQIP) Surgical Risk Calculator [7], might be helpful in considering the indication for surgery. However, anesthesiologists might not be fully aware of the patient’s or family’s personal wishes, making it difficult to solve this dilemma.

Given these circumstances, we created an institute-specific decision-making process for surgical treatment in 2010. These multidisciplinary preoperative conferences, termed High-Risk Conferences, aimed to optimize communication between care teams including physicians, nurses, technicians and medical social workers; allow for the determination of optimal treatment course; and promote sharing of treatment outcomes. The purpose of this study was to share the results of our High-Risk Conferences and to evaluate the effect of these conferences on patient outcomes.

## Methods

### High-risk conference process

**Fig. 1:**
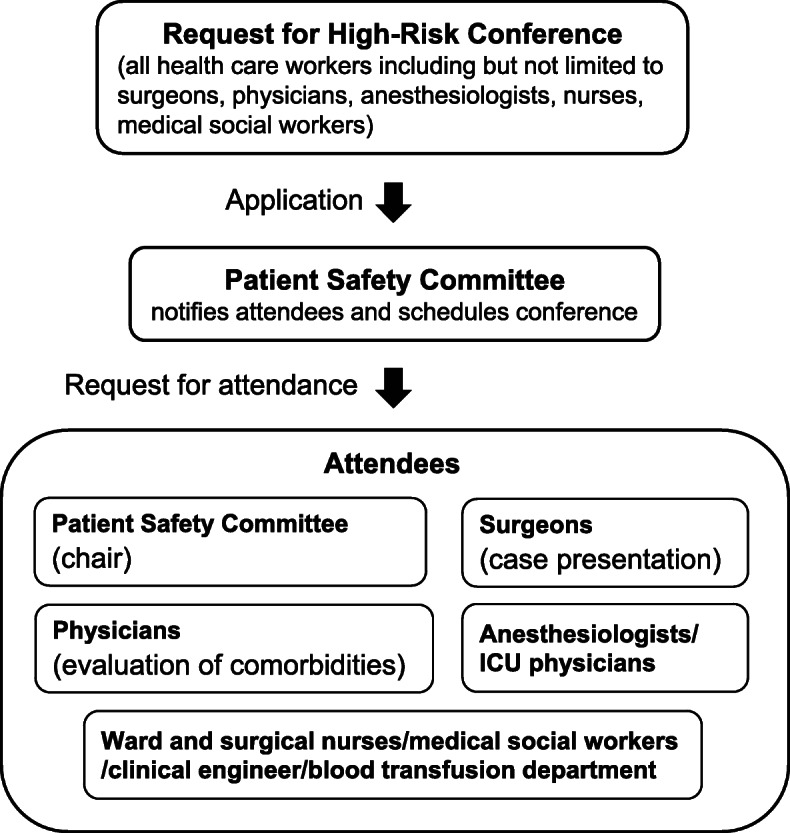
Flow chart of the decision-making process for multidisciplinary High-Risk Conferences. All health care professionals involved in patient care can request to convene a conference and attend. The attendees proceed with the conference on the basis of the initiative of the Patient Safety Committee, not that of the surgeon. The Patient Safety Committee is under the direct control of the director of our institution. The committee plays a central role in collecting and analyzing data regarding medical accidents/incidents, education/enlightenment activities related to patient safety and supporting the response to medical malpractice. ICU = intensive care unit

High-Risk Conferences were initiated at our institution in April 2010. These preoperative conferences involve the participation of multiple professionals of varying disciplines (Fig. 1). Conferences can be requested by any health care professional who perceives that there are medical or nonmedical/personal concerns regarding the course of treatment for the patient in question. The Patient Safety Committee coordinates the meeting schedules of attendees, and a member of the Patient Safety Committee serves as the chair. The conferences are generally held one to two weeks before the scheduled surgery date, which makes it possible to ensure sufficient time for preparation even if the treatment plan changes. Attendees include all health care professionals (e.g., physicians, nurses, clinical engineers, medical social workers). Physicians include surgeons and anesthesiologists as well as intensive care unit physicians and physicians assessing the patient’s comorbidities. Case presentations are made by surgeons, and participants discuss the appropriateness of the surgery given the patient’s medical and nonmedical/personal concerns and make efforts to draw conclusions that are best for the patient. These conferences proceed based on a group decision-making system and last approximately 30 to 40 min, depending on the issues discussed. The conclusions are based on the agreement of all participants. Multiple meetings are held for the same patient if a conclusion cannot be reached during a single meeting, and the decision at the last meeting is considered the final determination. Surgeons inform patients and their relatives of the conclusion as an institutional decision. For emergency surgeries, a High-Risk Conference with limited attendees can be held just before the procedure.

### Data collection

We retrospectively investigated proceedings, which were managed by the Patient Safety Committee, and patient medical records associated with High-Risk Conferences held from April 2010 to March 2018. Data collected included the number of meetings, patient characteristics, surgical departments involved, comorbidities, planned and actual surgical procedures and nonmedical/personal issues.

We assessed the reasons for convening conferences, categorizing them as (1) a need for a highly difficult surgery (novel and/or complex surgery), (2) the presence of severe comorbidity (ies) (whether patients can tolerate surgery and anesthesia) and (3) the presence of nonmedical/personal issues. The conclusions made during the conferences were evaluated and categorized as (1) surgery as planned, (2) modification of the surgical method and (3) surgery cancelled, with outcomes 1 year after surgery or conference investigated for patients for whom the surgical method was modified or the surgery was cancelled, respectively.

## Results

A total of 410 High-Risk Conferences were held for 406 patients during the study period (April 2010–March 2018), corresponding to 0.63% of the 64,762 anesthetic cases at our institution during this time period. A total of 34 conferences were held the first year. The number of meetings has increased in recent years to approximately 60 meetings per year. The clinical characteristics for the 406 patients discussed at the High-Risk Conferences are summarized in Table 1. A total of 53% of the patients were older than 65 years of age, and 40% had an American Society of Anesthesiologists (ASA) physical status of Class 3 or 4.

**Table 1:** Clinical characteristics of patients included in High-Risk Conferences (*N* = 406)

Characteristic	***n*** (%)	Characteristic	***n*** (%)
**Sex**		**Comorbidities for ASA physical status 3 and 4 (*n*** **= 161)**	
Male	240 (59.1)		
Female	166 (40.9)	Congestive heart failure	70 (43.5)
**Age, y**		Ventilator dependent	33 (20.5)
≤ 14	34 (8.4)	Hemodialysis	31 (19.3)
15–24	14 (3.4)	Dyspnea	26 (16.1)
25–34	25 (6.2)	Severe COPD	20 (12.4)
35–44	26 (6.4)	Disseminated cancer	18 (11.2)
45–54	40 (9.9)	Systemic sepsis	18 (11.2)
55–64	53 (13.1)	Ischemic heart disease	17 (10.6)
65–74	89 (21.9)	Acute renal failure	11 (6.8)
75–84	92 (22.7)	Diabetes	11 (6.8)
≥ 85	33 (8.1)	Stroke	5 (3.1)
**ASA physical status**		Other	30 (18.6)
1	30 (7.4)		
2	215 (53.0)		
3	122 (30.0)		
4	39 (9.6)		

*Abbreviations*: *ASA* American Society of Anesthesiologists, *COPD* chronic obstructive pulmonary disease

**Fig. 2:**
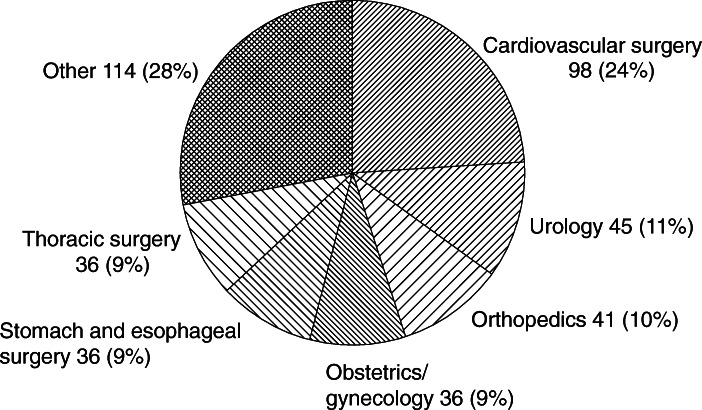
Breakdown of the clinical departments responsible for cases discussed at High-Risk Conferences. Data are presented as number of cases (%). Cardiovascular surgery, which conducts highly difficult and advanced surgeries for patients with severe comorbidities, had the greatest number of conferences (24%). Urology had 11%, leading in surgical procedures, such as those for huge renal cell carcinoma or retroperitoneal tumors, performed across multiple surgical departments. Conferences for orthopedics were generally focused on the patient’s ability to tolerate the surgery and anesthesia

**Fig. 3:**
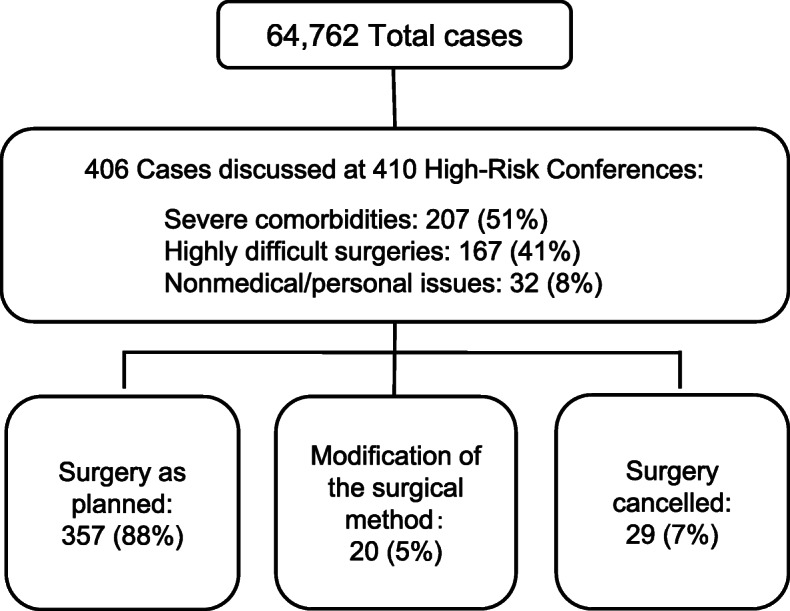
Reasons for convening and conclusions determined at High-Risk Conferences. The presence of severe comorbidities and whether patients with could tolerate surgery and anesthesia was the most common reason for convening a conference. After a High-Risk Conference, surgery was conducted as planned for 88% of cases. Changes in treatment plan occurred for 12% (5% surgical procedure modified and 7% surgery cancelled)

A breakdown of High-Risk Conferences by clinical department is shown in Fig. 2. Cardiovascular surgery had the greatest number of cases (98 cases, 24%), followed by urology (45 cases, 11%) and orthopedics (41 cases, 10%). The reasons for convening and conclusions made at the High-Risk Conferences are shown in Fig. 3. The most common reason for a conference was to discuss whether a patient with severe comorbidities could tolerate surgery and the invasiveness of anesthesia (207 conferences, 51%). A total of 167 conferences (41%) were held to discuss whether to conduct rarely performed, highly difficult, advanced or complex surgeries spanning multiple departments or to share information on these surgeries. A total of 32 conferences (8%) were held because of nonmedical/personal issues. There were cases in which a patient with an expected survival of less than a few months had conveyed do-not-resuscitate orders, an infant for whom parental neglect was an issue and a patient for whom it was necessary to check their willingness to undergo surgery because of poorly controlled schizophrenia. For most of the patients, there was more than one reason to hold a conference. For example, it was often necessary to evaluate whether a patient with severe cardiovascular or pulmonary comorbidities would be exposed to an unacceptable risk of serious perioperative complications after highly difficult surgery. As a result of discussions at these conferences, 357 surgical procedures (88%) were performed as planned. For 49 cases (12%), the treatment plan was changed, with 20 resulting in modification of the surgical procedure, and 29 resulting in cancellation of the surgery.

Clinical data for the 20 cases for which conferences resulted in a modified procedure are shown in Table 2. Modifications to the surgical procedure were classified as (1) a reduction in the procedure or a switch to palliative surgery after considering the difficulty of the surgical procedure or the patient’s condition (e.g., severe medical comorbidities) (16 cases), (2) a change in surgery priority for patients requiring multiple surgeries (2 cases) and (3) a change to a more expansive surgery with the aim of radical treatment (2 cases). Four patients in the modification group died, all of whom underwent procedures converted to palliative surgery, and all four died within four months of surgery.

**Table 2:** Clinical data for 20 modified cases

No.	Age/sex	Diagnosis	Planned surgery	Surgery performed (reason for modification)	Outcome one year after surgery
1	51/M	Oesophageal cancer, aortic invasion, mediastinitis	Esophagectomy, aortic replacement	Esophagectomy, intercostal muscle flap to aorta (R)	Survived
2	73/F	Lumbar spinal stenosis, diabetic gangrene, CKD on HD, CAD	Laminectomy, lower limb amputation	Lower limb amputation (R)	Survived
3	80/F	Ureteral-right iliac artery fistula, common iliac artery aneurysm	Iliac artery replacement by laparotomy	Stent graft (R)	Survived
4	46/M	Pancreatic cancer, pheochromocytoma	PD	Laparoscopic right adrenalectomy (P)	Survived, PD after one month
5	62/M	Salivary fistula and ileostomy after esophagectomy	Esophageal reconstruction by thoracotomy	Pectoralis major myocutaneous flap to prevent salivary fistula (R)	Survived
6	7/F	Tumor in left arm	Biopsy, Broviac catheter insertion, bone marrow aspiration	Biopsy (R)	Survived
7	63/M	Graft infection after aortic replacement for TAAA	Removal of graft on CPB by median sternotomy	Removal of graft without CPB by thoracotomy (R)	Survived
8	78/F	Loosening after total hip arthroplasty with screw into the pelvis	Removal of implant, debridement	Removal of implant, debridement, colostomy, sigmoidectomy (E)	Survived
9	74/M	TAAA	Aortic replacement by thoracotomy and laparotomy	Aortic replacement by laparotomy (R)	Survived
10	84/M	Bladder cancer	Laparoscopic cystectomy, ureteral fistula	Ureteral fistula (R)	Died of infection POD 25
11	66/M	Gastric cancer	Gastric wedge resection	Distal partial gastrectomy (E)	Survived
12	63/M	Liver cirrhosis, liver cancer	Radiofrequency ablation by laparotomy	Percutaneous radiofrequency ablation (R)	Survived
13	31/M	Lung injury caused by chest tube, muscular dystrophy complicated with cardiomyopathy	Repair by thoracotomy	Bronchoscopic embolization (R)	Died of heart failure POD 2
14	66/F	Aortic valve stenosis, mitral valve regurgitation, post CABG	Double valve replacement	Aortic valve replacement (R)	Died of heart failure POD 38
15	86/M	Gastric cancer, CKD on HD, paraplegia after stent grafting for TAA	Distal partial gastrectomy	Gastric wedge resection (R)	Died of heart failure POD 106
16	60/F	Lung tumor, aortic invasion	Lower lobectomy, aortic replacement	Thoracoscopic biopsy (R)	Chemotherapy, survived
17	59/M	Esophageal tumor, tracheal invasion	Esophagectomy, combined resection of trachea	Biopsy (R)	Chemotherapy and radiotherapy, survived
18	71/M	Esophageal cancer, liver cancer	Esophagectomy, partial hepatectomy	Esophagectomy (R)	Chemotherapy for liver cancer, survived
19	1/M	Functional pyloric stenosis after necrotizing enterocolitis	Pyloroplasty, closure of ileostomy, colostomy	Ileotransversostomy (R)	Survived
20	70/M	Gallbladder cancer, mitral valve regurgitation, tricuspid valve regurgitation	Extended cholecystectomy	Mitral valve replacement, tricuspid valve replacement (P)	Extended cholecystectomy at 20 months, survived

*Abbreviations*: *CABG* coronary artery bypass graft, *CAD* coronary artery disease, *CKD* chronic kidney disease, *CPB* cardiopulmonary bypass, *E* changed to more expansive surgery for radical treatment, *HD* hemodialysis, *P* changed to priority surgery due to patient’s clinical condition, *PD* pancreaticoduodenectomy, *POD* postoperative day, *R* reduction in the procedures or changed to palliative surgery due to surgical difficulties or patient comorbidities, *TAA* thoracic aortic aneurysm, *TAAA* thoracoabdominal aortic aneurysm

For the 29 conferences during which cancellation of surgery was decided, the most common reason was that patients had severe cardiovascular or pulmonary comorbidities, with 19 patients deemed unable to tolerate surgery. Some of the patients for whom surgery was cancelled were also transferred to other institutions. We were able to follow up 21 of these 29 patients one year after the conference. Clinical data for these 21 patients are shown in Table 3. A total of 11 patients died; 6 of their primary disease and 5 of comorbidities. Eight died within one month of the conference.

**Table 3:** Clinical data for 21 cancelled cases followed up after one year

No.	Age/sex	Diagnosis	Planned surgery	Reason(s) for cancellation	Outcome after one year
1	87/F	Ileus	Gastrojejunal bypass	Terminal stage, peritoneal carcinomatosis, aspiration pneumonia	Died of cancer 99 days after the conference
2	54/F	Brain tumor, SLE	Resection	Severe infection due to long-term steroid administration	Survived
3	74/F	Ileus	Metallic stent insertion	Terminal stage, peritoneal carcinomatosis, liver failure	Died of cancer 24 days after the conference
4	69/M	Lung cancer, empyema with bronchopleural fistula	Musculocutaneous flaps plombage	Respiratory failure	Died of respiratory failure 13 days after the conference
5	64/F	Pancreatic cancer, SLE	PD	Pancreatitis, CKD on HD	Died of cancer 121 days after the conference
6	70/M	Oesophageal cancer	Esophagectomy	COPD on HOT	Radiotherapy and chemotherapy, survived
7	68/F	Hepatic hemangioma	Resection	CKD on HD	Survived
8	56/F	Metastatic ovarian cancer	BSO	Surgical difficulty, rapidly growing cancer	Died of cancer 19 days after the conference
9	80/M	Gangrene of the lower limb	Amputation	Low cardiac function, COPD, CKD on HD	Died of heart failure four days after the conference
10	46/M	Liver failure	Living donor liver transplant	Ethical issue (family refusal)	Died of liver failure the day after the conference
11	1/M	Liver tumor	Open biopsy	Low birth weight, tracheomalacia, chronic lung disease	Survived
12	72/M	Lung cancer	Partial pulmonary resection	COPD	Chemotherapy, survived
13	79/M	Gastric cancer	Complete gastrectomy	CKD on HD, carotid artery stenosis	Chemotherapy, survived
14	43/F	Large retroperitoneal tumor	Resection	Surgical difficulty	Chemotherapy, survived
15	72/M	Renal-cell carcinoma	Laparoscopic nephrectomy	Pneumonia, heart failure	Died of pneumonia 19 days after conference
16	1/M	Pilomyxoid astrocytoma	Ventriculoperitoneal shunt	Ethical issue (patient in vegetative state)	Radiotherapy, survived
17	63/F	Spondylolisthesis	Spinal fusion	LC, CKD on HD, PE	Survived
18	79/M	Gastric cancer	Complete gastrectomy	COPD, AS, CAD	Died of MI 23 days after the conference
19	76/M	Large retroperitoneal tumor	Resection	Surgical difficulty	Chemotherapy, survived
20	34/M	Cervical spine injury, AIDS	Internal fixation	Severe infection	Died of pneumonia seven days after the conference
21	68/M	Lung cancer, bronchopleural fistula	Closure	COPD	Died of cancer 134 days after the conference

*Abbreviations*: *AIDS* acquired immunodeficiency syndrome, *AS* aortic valve stenosis, *BSO* bilateral salpingo-oophorectomy, *CAD* coronary artery disease, *CKD* chronic kidney disease, *COPD* chronic obstructive pulmonary disease, *HD* hemodialysis, *HOT* home oxygen therapy, *LC* liver cirrhosis, *MI* myocardial infarction, *PD* pancreaticoduodenectomy, *PE* pulmonary embolism, *SLE* systemic lupus erythematosus

## Discussion

We examined the details of 410 multidisciplinary High-Risk Conferences conducted at our institution over an 8-year period. Approximately half of the patients discussed at the conferences were aged 65 years or older and had an ASA physical status of Class 3 or 4. The most common reason for convening a conference was to evaluate the ability of patients with severe comorbidities to tolerate surgery. As a result of these conferences, changes to treatment plans, including modification of the surgical method or surgery cancellation, were made for 12% of the patients.

Having discussions in advance for patients with complex issues is widely practiced. Numerous medical institutions have introduced systems, such as preoperative outpatient clinics and consultations with anesthesiology departments, to enhance perioperative safety [5, 8]. However, these discussions are usually only between physicians. The High-Risk Conference system reported here involves numerous health care professionals of varying disciplines, including nurses, technicians and medical social workers as well as physicians, all of whom share patient information from different perspectives and discuss fundamental content. Conclusions are based on a group decision-making system, resulting in a change in the treatment plan for 12% of the patients in the present study. We believe that this group decision-making system provides patients with the best course of treatment, taking into account various factors and clinical perspectives.

A survey from the United Kingdom reported a mortality rate of 12.3% in a group of patients undergoing high-risk surgery [9]. In our study, the mortality rate in the first year after surgery was 20% in the modification group and 52% in the cancellation group (of those who could be followed up), showing the extreme fragility of patients discussed at these conferences. Indeed, 40% of patients discussed at the conferences were ASA Class 3 or 4. The main reason for cancelling surgery or modifying the surgical method was the patient’s inability to tolerate surgery, and many of the surgical modifications were conversions to less-extensive procedures. We assume that the incidence of perioperative death and complications would be greater if these patients had undergone the surgeries as planned. None of the cases discussed at High-Risk Conferences resulted in medical lawsuits from patients or their families.

Before surgery, an anesthesiologist might occasionally feel that the operation will be in vain. Indeed, there are many instances of patients with numerous severe comorbidities who have undergone prolonged procedures that do not result in the outcome the patient or family expected. Because anesthesiologists come into contact with patients and families less frequently than surgeons do, it is difficult for anesthesiologists alone to argue that surgery might not be appropriate. However, a High-Risk Conference, based on group decision making across multiple disciplines including nursing and medical social work, can evaluate medical as well as nonmedical/personal issues and decide whether to perform surgery. In the present study, among patients for whom surgery was cancelled, eight died within one month of the conference, and five died as a result of comorbidities, showing that the conference may have allowed us to avoid surgery that might have ended in vain. Avoiding surgeries that could lead to unfavorable outcomes might also result in more effective use of limited medical resources [10].

As a result of the High-Risk Conferences at our institution, surgery was performed as planned for 88% of the patients in the present study. Looking at this finding alone, it might be inferred that these conferences are a waste of time. However, one of the advantages of High-Risk Conferences is the ability to tailor management strategies during the entire perioperative period in advance. Internal medicine physicians, anesthesiologists and intensive care physicians can discuss, share and agree on policies of perioperative management for patients with severe comorbidities at a single meeting. In addition, as shown in Figs. 3, 41% of patients discussed at these High-Risk Conferences required complex or advanced novel procedures as well as procedures performed across several surgical departments. In these cases, information, such as how to use devices unfamiliar to nonsurgeons, deployment of equipment into the operating room, the order of the procedure and the time required when multiple departments are involved, can be shared across multiple professionals. In general, these highly difficult surgeries are associated with a high likelihood of massive bleeding. Preparedness for blood transfusion on the day of surgery can also be agreed upon in advance by adding staff from the blood transfusion department to these High-Risk Conferences as necessary. Moreover, even if the decision to perform the surgery as scheduled is made at a High-Risk Conference, the conditions for withdrawal after initiation of surgery are determined for some patients. Predetermining withdrawal conditions prevents delayed decision making and inadvertent exposure of patients to dangerous conditions for prolonged periods of time. Thus, High-risk Conferences that enable information to be shared in advance have contributed to a smooth and safe process even when surgery is performed as planned. As shown in Table 1, 60% of patients discussed at the conferences had an ASA physical status of Class 1 or 2. For most types of high-risk conferences, only medical comorbidities are discussed, and patients with ASA physical status Class 1 or 2 are generally not included. One of the characteristics of our High-Risk Conferences is that the processes for difficult or advanced surgeries, as well as nonmedical/personal issues, are discussed for patients with ASA physical status Class 1 or 2.

Since mid-2010, it has been recommended that these types of conferences be held in situations involving challenging surgical treatment options [11]. At our institution, the utility of decision making across multiple disciplines was quickly recognized, and High-Risk Conferences have been held since April 2010. Several reports on high-risk conferences have been published [12–14]; however, they appeared to focus more on process. We analyzed outcomes for numerous patients after High-Risk Conferences, particularly those for whom procedures were modified or cancelled. Sroka et al. reported on the utility of high-risk conferences at a cancer center [14]. In that study, discussions of medical comorbidities were the main focus, and anesthesiologists usually took the initiative at the conferences. In fact, anesthesiologists play an important role in multidisciplinary surgical teams during the perioperative period because they are used to evaluating patients from the many points of view [15]. At our institution, all relevant parties/health care professionals, including nonphysicians, may request assembly and participation, and the meetings are run by a neutral party (Patient Safety Committee). Thus, the system allows for an overarching, neutral discussion with knowledge exchange on topics including surgical procedures and nonmedical/personal issues for the patient rather than just a discussion of comorbidities.

### Study limitations

High-Risk Conferences at our institution are continued until one of the following conclusions is reached: surgery as planned, modification of the surgical method or surgery cancelled. This decision is the final decision of the institution, and even the attending physician cannot overturn the decision on their own. For 12% of patients in the present study, a High-Risk Conference resulted in conclusions that differed from the initial judgment of the attending physicians. The validity of the conclusions drawn at these conferences needs to be investigated, but the clinical data for the patients evaluated were very complex. Novel as well as complicated or rare surgical methods are subjects of discussion at High-Risk Conferences. It is difficult to compare these patient data and surgical methods. Statistical methods are unsuitable for evaluating the validity of conducting High-Risk Conferences. We also did not use a definition for high-risk surgical patient or clarify criteria for holding conferences. However, any health care professional can request a High-Risk Conference when they have concerns regarding the course of treatment for a patient in our system. Although this process provides opportunities to discuss various issues, it is possible that we did not hold all conferences necessary.

## Conclusions

Our assessment of High-Risk Conferences showed that the conferences were not just endorsements of treatment policies set by surgeons. Most of the changes in the treatment plan were changes to less-extensive procedures or cancellation because of issues with the patient’s ability to tolerate surgery. These changes might have decreased perioperative mortality and complications as well as unnecessary surgeries. Organizing more conferences will consume extra time and resources, and it may also entail changes to the cultural habits and norms of surgeons [16]. However, the number of meetings has gradually increased over the years at our institution, possibly because the health care professionals involved have realized the utility of these conferences. We believe that High-Risk Conferences contribute to the smooth and safe execution of surgeries at our institution.

## Data Availability

The datasets used and analyzed during the present study are available from the corresponding author on reasonable request.

## Abbreviations

ACS NSQIP: American College of Surgeons National Surgical Quality Improvement Program
AIDS: Acquired immunodeficiency syndrome
AS: Aortic valve stenosis
ASA: American Society of Anesthesiologists
BSO: Bilateral salpingo-oophorectomy
CABG: Coronary artery bypass grafting
CAD: Coronary artery disease
CKD: Chronic kidney disease
COPD: Chronic obstructive pulmonary disease
CPB: Cardiopulmonary bypass
E: changed to more expansive surgery for radical treatment
HD: Hemodialysis
HOT: Home oxygen therapy
ICU: Intensive care unit
LC: Liver cirrhosis
MI: Myocardial infarction
P: Changed to priority surgery due to patient’s clinical condition
PD: Pancreaticoduodenectomy
PE: Pulmonary embolism
POD: Postoperative day
R: Reduction in the procedures or changed to palliative surgery due to surgical difficulties or patient comorbidities
SLE: Systemic lupus erythematosus
TAA: Thoracic aortic aneurysm
TAAA: Thoracoabdominal aortic aneurysm
